# Dietary Nitrate and the Epidemiology of Cardiovascular Disease: Report From a National Heart, Lung, and Blood Institute Workshop

**DOI:** 10.1161/JAHA.116.003402

**Published:** 2016-07-06

**Authors:** Amrita Ahluwalia, Mark Gladwin, Gary D. Coleman, Norman Hord, George Howard, Daniel B. Kim‐Shapiro, Martin Lajous, Filip J. Larsen, David J. Lefer, Leslie A. McClure, Bernard T. Nolan, Ryszard Pluta, Alan Schechter, Chia‐Yih Wang, Mary H. Ward, Jane L. Harman

**Affiliations:** ^1^ The William Harvey Research Institute, Barts & The London Medical SchoolQueen Mary University of LondonUK; ^2^ Vascular Medicine InstitutePittsburgh UniversityPittsburghPA; ^3^ University of MarylandCollege ParkMD; ^4^ Oregon State UniversityCorvallisOR; ^5^ University of Alabama at BirminghamBirminghamAL; ^6^ Wake Forest UniversityWinston‐SalemNC; ^7^ Nacional de Salud Pública de MexicoMexicoAlbania; ^8^ Karolinska InstituteStockholmSweden; ^9^ Louisiana State University Health Sciences CenterNew OrleansLA; ^10^ Dornsife School of Public Health at Drexel UniversityPhiladelphiaPA; ^11^ US Geological SurveyRestonVA; ^12^ National Institute of Neurological Disorders and StrokeBethesdaMD; ^13^ National Institute for Diabetes and Digestive and Kidney DiseasesBethesdaMD; ^14^ National Center for Health Statistics, CDCHyattsvilleMD; ^15^ National Cancer InstituteRockvilleMD; ^16^ Division of Cardiovascular SciencesNational Heart, Lung, and Blood InstituteBethesdaMD

**Keywords:** national registry, nitrate, nitric oxide, nutrition, Endothelium/Vascular Type/Nitric Oxide, Pathophysiology

## Introduction

In view of continuing unanswered questions regarding the geographical and demographic distribution of cardiovascular disease, and recent discoveries about the effects of dietary nitrate on cardiovascular physiology, the National Heart, Lung, and Blood Institute (NHLBI) convened a workshop to identify approaches to address how best to incorporate the study of nitrate exposures into ongoing studies of cardiovascular epidemiology. The NHLBI invited speakers who had made recent contributions to the study of the functions of nitrate on the cardiovascular system, on the occurrence of nitrate in foods and drinking water, or who had expert knowledge of cardiovascular surveys with wide geographical variability and therefore the greatest potential variability in dietary and drinking water nitrate. Because of the history of research on the possible carcinogenicity of nitrite, an expert in this field was also invited. The following document is a synthesis of the material presented and discussed and of literature cited at the workshop. The workshop from which this article is derived was funded and convened by the NHLBI.

## From Dietary Nitrate to Nitric Oxide

Nitrate (NO_3_^−^) is an essential plant nutrient found in soil after the fixation of atmospheric nitrogen by the action of lightning or soil microbes. As a component of plants, nitrate enters the human diet mainly through the consumption of vegetables whereas nitrite (NO_2_^−^) enters the diet through consumption of processed foodstuffs, particularly resulting from use in meat preservation.^1^ Dietary nitrate and nitrite confer physiological effects that have been observed by physicians for over 2000 years, and which have now been attributed to the action of nitric oxide (NO) through the recently discovered endogenous nitrate‐nitrite‐nitric oxide pathway.^2^ Nitric oxide was identified as an important biologically active molecule in the late 1980s as the elusive “endothelium‐derived relaxation factor.” Soon thereafter, nitric oxide was recognized as a signaling molecule involved in a vast number of physiologic processes, including regulation of blood flow and blood pressure. Nitric oxide–mediated signaling is also used to protect the heart against cellular injury or death and helps regulate mitochondrial respiration by its reversible inhibition of cytochrome c oxidase.

For many years after the discovery of nitric oxide's biological role, it was assumed that the body's supply of nitric oxide was produced solely by the action of nitric oxide synthase on the amino acid, l‐arginine, so that dietary protein was the ultimate source of nitric oxide. The production of nitric oxide from l‐arginine requires molecular oxygen and a number of other critical factors, including tetrahydrobiopterin. Nitric oxide is rapidly oxidized to nitrite and nitrate, 2 reaction products that were thought to be metabolically inactive and destined only for excretion in the urine. These assumptions were wrong on 2 counts: Although oxidation of nitric oxide to nitrate and nitrite acutely terminates nitric oxide bioactivity, it is now recognized that there is a pathway that recycles these anions back into nitric oxide within the body,^3^ providing another important source. Because this pathway uses nitrate to produce nitrite and then nitric oxide, we now know that dietary nitrate, obtained mostly through dietary vegetable content, is an important source of the body's supply of nitric oxide. And very importantly, this pathway for producing nitric oxide from the reduction of nitrite does not require oxygen and is critical under conditions of relative hypoxia, when the vasodilatory effect of nitric oxide is most needed.

## Nitrate Absorption and Enterosalivary Reduction Cycle

Nitrate occurs in the diet mostly as a component of green leafy and root vegetables. After the ingestion of a nitrate‐rich meal, this anion is almost 100% absorbed through the gastrointestinal tract. Nitrate is then extracted from the circulation by the salivary glands and actively secreted into the saliva, such that salivary nitrate concentration may be greater than 10‐fold that found in plasma.^4^ Analogous to the action of bacteria in the soil, bacteria in the oral cavity employ this salivary nitrate in their respiration pathway, using intrinsic nitrate reductase enzymes to reduce it to nitrite.^5^ Higher‐order animals are not capable of reducing nitrate to nitrite, so this crucial chemical reduction step by the oral microbiome is a marvelous example of vertebrate‐bacteria commensalism. The resulting microbial‐produced nitrite is swallowed, whereupon much of it is absorbed through the gastrointestinal tract as nitrite and some of it is reduced to nitric oxide in the stomach; the nitric oxide so produced contributes to gastric mucosa integrity and provides protection against colonization of the stomach by infectious agents.^6^, ^7^, ^8^

Plasma nitrate levels rise immediately after a nitrate‐rich meal, with a half‐life of ≈5 hours, and plasma nitrite levels subsequently rise in parallel, slightly delayed by the enterosalivary cycling for the reduction of nitrate to nitrite.^9^, ^10^ Practitioners of ancient Chinese medical arts demonstrated empirical knowledge of this enterosalivary cycle: These healers wrote that while administering saltpeter (potassium nitrate) for heart pain, it is important for the patient to swallow any saliva that is produced.^2^ It has also been demonstrated that the salivary reduction of nitrate to nitrite can be inhibited by antiseptic mouthwash, which kills the necessary tongue microorganisms; the formation of nitric oxide in the stomach can also be lowered by the use of proton pump inhibitors, which diminish stomach acidity.^11^, ^12^

In the stomach, some of the swallowed nitrite can alternatively be protonated to HNO_2_, which, in turn, spontaneously yields dinitrogen trioxide (N_2_O_3_), nitrogen dioxide (NO_2_), and nitric oxide (NO). The N_2_O_3_ so formed is a powerful nitrosating agent capable of donating a nitrosonium cation (NO^+^) to secondary and tertiary amines to form potentially carcinogenic *N*‐nitrosamines.^13^ Under ingestion conditions lacking food components that protect against nitrosation reactions, HNO_2_ can be protonated to H_2_NO_2_, which reacts with amides to form *N*‐nitrosamides. These mechanisms of endogenous nitrosation may contribute substantially to human exposure to N‐nitroso compounds.^14^

## The Production of Nitric Oxide From Circulating Nitrite

Most of the swallowed salivary nitrite is absorbed in the gastrointestinal tract and enters the circulation, becoming available to all tissues of the body. Nitrite is now available for reduction to the active messenger, nitric oxide, especially under conditions of physiological or pathological hypoxia, when nitric oxide cannot be produced from l‐arginine by nitric oxide synthases. The nitrite anion can be considered a circulating storage pool for nitric oxide bioactivity^15^ that regulates hypoxic vasodilation^16^ and the cellular resilience to low oxygen and ischemia.^17^

In addition to their roles as oxygen transporters, hemoglobin and myoglobin function as allosterically regulated nitrite reductases under hypoxic conditions.^16^, ^18^, ^19^ Under conditions of hypoxia, blood nitrite reacts with deoxyhemoglobin to generate nitric oxide to cause vasodilation. Deoxymyoglobin serves a similar function in muscle. This effective nitrite reductase activity in the vascular space by red cells comprises an important component of the body's overall nitric oxide production. In the heart, nitric oxide availability by the nitric oxide synthase pathway is induced by exercise training; however, the increase in nitric oxide synthase is also associated with increased levels of myocardial nitrite storage. During subsequent periods of hypoxia, a condition under which nitric oxide synthase activity is inhibited, this stored nitrite is available for rapid reduction to nitric oxide to protect the heart from myocardial ischemia,^20^ and deoxymyoglobin has been implicated as a key nitrite reductase involved in this process.^21^

A number of cellular enzymes/proteins regulate nitrite reduction to nitric oxide at different oxygen tensions and with organ system specificity. It is proposed that the nitrate‐nitrite‐nitric oxide pathway represents a fundamentally conserved pathway for energetics and signaling in biology. The role of molybdenum‐containing enzymes and heme‐globin superfamily proteins seem to be especially important as nitrite reductases: xanthine oxidoreductase, aldehyde oxidase, mitochondrial amidoxime reductase, and sulphite oxidase; the four mammalian molybedenum containing enzymes have all individually been shown to possess significant nitrite reductase activity.^22^, ^23^, ^24^ Similarly, all of the mammalian globins, hemoglobin, myoglobin, neuroglobin, cytoglobin, and the plant and *Drosophila* hemoglobin, are the subject of active current study.^25^ Studies of cytochrome C, neuroglobin, and plant hemoglobins have identified a role for heme coordination in the control of nitrite reduction to nitric oxide (ie, 6‐to‐5 coordinate regulation of nitrite binding and reduction).^25^ Interestingly, the predominance of these two key, but distinct, types of nitrite reductase seems to depend upon whether the activity relates to nitrite reduction in health (globins) or nitrite reduction in disease (xanthine oxidoreductase).^26^

## Physiological Effects of Exogenous Nitrate and Nitrite

Because nitrate and its reduction product, nitrite, are reservoirs for nitric oxide production, many of the very important roles played by nitric oxide—including its antithrombotic and immune modulatory effect and its role in cytoprotection and vasodilation—have now been shown to be effects likewise occurring with provision of exogenous sources of nitrate and nitrite. In animal models of myocardial infarction,^17^, ^27^ heart failure,^28^ pulmonary hypertension,^29^, ^30^, ^31^ and vascular hypertrophy,^32^ dietary provision of nitrate or nitrite improves outcome in terms of infarct size, cardiac function and hypertrophy, pulmonary arterial pressures, and cardiac and vascular hypertrophy, respectively.

Perhaps the most well‐reported and consistent observation with oral nitrate ingestion is that it lowers blood pressure. The first demonstration of this phenomenon came from a study using sodium nitrate salt solution to deliver nitrate in 17 healthy volunteers, in whom significant reductions in diastolic blood pressure (3.5 mm Hg) were evident with a dose that approximated a nitrate‐rich vegetable meal, such as a lettuce salad.^33^ In 2008, it was demonstrated that ingestion of nitrate‐rich beetroot juice in 14 healthy volunteers caused decreases in both systolic (≈10 mm Hg) and diastolic (≈8 mm Hg) pressures.^34^ Further studies demonstrated a dose‐response relationship in 19 healthy volunteers with a 4‐mmol dose representing a threshold dose with little blood pressure (BP)‐lowering effect. In contrast, in 14 patients with hypertension, delivery of just below this threshold dose (3.5 mmol) caused BP lowering similar in magnitude to that which might be achieved with antihypertensive pharmacotherapy (≈12/8 mm Hg), intimating possibly an increased potency in disease scenarios. Recent studies in 64 hypertensive patients both drug naïve and those on multiple medications demonstrate that the effects of a once‐daily dose are sustained over the long term. In this study, a single dose of dietary nitrate (beetroot juice) was consumed once‐daily for 4 weeks and the BP‐lowering effects sustained for the duration of ingestion.^35^ Whereas these studies together suggest clinically relevant levels of BP lowering in both healthy volunteers and hypertensive patients, further studies both of longer duration and in larger cohorts to determine the general clinical translatability across the diverse hypertensive patient profile would be of value.

Ingestion of inorganic nitrate as a supplement has also captured the attention of the sports and exercise fraternity. Administration of inorganic nitrate increases the efficiency of oxidative metabolism, evident as reduced oxygen consumption, whether the body is at rest or at maximal exertion of large muscle groups.^36^, ^37^, ^38^ This effect is coupled to enhanced mitochondrial respiratory efficiency in human skeletal muscle and a reduced proton leak across the inner mitochondrial membrane.^39^ The reduction in metabolic rate seems to be strongly influenced by the active uptake of nitrate in saliva.^37^ Recently, based on studies showing improvement in the efficiency of oxidative metabolism, there has been a proliferation of sports supplements containing high amounts of nitrate that claim to boost athletic performance.^40^

Dietary supplementation with nitrite also inhibits platelet reactivity and increases bleeding time, and as such, the nitrate‐nitrite‐nitric oxide pathway may provide a way to modulate blood clotting processes pharmacologically. Nitric oxide inhibits aggregation of platelets and, as shown recently,^41^ reduction of nitrite at 0.1 μmol/L by red cells consequently inhibits platelet aggregation and ATP release, decreases P‐selectin, and increases cGMP levels in human platelets ex vivo with various agonists, such as ADP and collagen. This inhibitory effect of nitrite on platelet aggregation is enhanced by deoxygenation of the red cells; this inhibition of platelet aggregation is, in turn, blocked by a nitric oxide inhibitor. Because erythrocytic hemoglobin must be partially deoxygenated in order to reduce nitrite to NO, this suggests a basis for the known differences in arterial and venous blood clotting. In murine models, platelet reactivity is inversely correlated with plasma levels of these 2 anions, especially nitrite levels. Dietary nitrate administration to healthy human subjects results in a reduction in platelet reactivity assessed ex vivo in response to ADP and collagen,^42^ an effect that is critically dependent upon the enterosalivary circuit and elevation of circulating nitrite levels.^34^

Exogenous nitrite as a source of nitric oxide has recently emerged as a promising therapy to attenuate myocardial injury and improve cardiac performance in the setting of heart failure. In most cardiovascular diseases, including heart failure, endothelial nitric oxide synthase activity is significantly attenuated, resulting in depletion of both nitric oxide and nitrite within the myocardium; and this loss of nitric oxide–mediated signaling contributes to the pathogenesis of acute myocardial infarction and heart failure. However, it has been demonstrated that oral administration of sodium nitrite protects mice against myocardial ischemia‐reperfusion injury, as well as against chronic cardiac hypertrophy and heart failure in mice subjected to pressure overload. Infused nitrite similarly protected mice against both hepatic and cardiac ischemia‐reperfusion injury.^17^ More recently, efforts to translate these latter observations to the clinical setting have produced mixed results in patients presenting with ST‐elevated myocardial infarction. Intravenous infusion of sodium nitrite before primary percutaneous coronary intervention (PPCI) proffered no benefit in terms of infarct size,^43^ but intracoronary administration^44^ resulted in reductions in infarct size in a subgroup of patients classified with an occluded culprit artery at the time of PPCI.

With respect to heart failure, very recent research indicates positive effects of dietary nitrate, through beetroot juice delivery, on exercise capacity^45^ and endurance^46^ effects that may be related to improvements specifically in skeletal muscle activity.^47^ In a related manner, evidence suggests potential for the nitrate‐nitrite‐nitric oxide pathway in the therapeutics of pulmonary hypertension. Indeed, inhaled nitrite reverses hypoxic neonatal pulmonary hypertension in sheep^48^ and dietary nitrite and nitrate exert similar effects in mice with pulmonary hypertension induced either by hypoxia or bleomycin.^30^ In humans, inhaled and oral nitrite may be able to prevent and reverse established pulmonary arterial hypertension^29^; phase 2 proof‐of‐concept trials for this indication are currently in progress in the United States and Europe.

The effects of nitrate‐rich beetroot juice on aging of the human cardiovascular system has been investigated. A high nitrate diet in older persons did not alter global cerebral perfusion, but did lead to increased regional cerebral perfusion in frontal lobe white matter.^49^ In older chronic obstructive pulmonary disease patients given beetroot juice, exercise time at constant work load was significantly longer compared to those given placebo.^50^ However, a significant lowering of BP was not observed in older adults with controlled hypertension undergoing supervised exercise therapy while consuming a high‐nitrate beverage compared to those on supervised exercise consuming a placebo beverage. All subjects underwent supervised exercise training 3 times per week for 6 weeks. Exercise lowered BP and improved other vascular measures, but dietary nitrate had no additional beneficial effects.^51^

Exogenous administration of nitrite may have a role in attenuation of the ischemic injury to the brain poststroke. Since its discovery, nitric oxide has captured the attention of neuroscientists because of its many roles in the brain: the regulation of brain blood flow,^52^, ^53^ the inhibition of platelet activation as a crucial event in cerebral embolism, cerebral ischemia,^54^ mediation of inflammatory response,^55^ limitation of reperfusion injury,^56^ activity as a reactive oxygen species scavenger,^57^ role in synapse‐less neurotransmission,^58^ and as a regulator of blood–brain barrier permeability in brain tumors.^59^ Early investigations focused on the presence and activity of various nitric oxide synthase enzymes in the brain.^60^ Subsequent to recent elucidation of endogenous nitric oxide production pathways, interest has shifted toward brain‐specific effects of reduction of nitrite to nitric oxide that depends on local hypoxia^61^ and the presence of deoxygenated hemoglobin, neuroglobin,^62^, ^63^ and other enzymes.^64^ Several seminal studies have confirmed the feasibility of exogenous nitrite or nitrate as a physiological source of nitric oxide to limit ischemic damage to brain after stroke and reperfusion.^16^, ^65^, ^66^, ^67^, ^68^

## Dietary Nitrate and Potential Cardiovascular Benefits

The aforementioned trials demonstrating beneficial effects of exogenous nitrate and nitrite suggest that a habitual ingestion of high‐nitrate‐containing foods may have beneficial effects on cardiovascular disease risk. Humans are exposed to nitrate on a daily basis through their diet because vegetables are a rich source of nitrate, with especially high amounts of nitrate found in green leafy vegetables and root vegetables. Accordingly, studies have demonstrated efficacy of oral nitrate delivered through dietary components, such as beetroots, as demonstrated in the experiments described previously.

Epidemiological studies have consistently found that fruit‐and‐vegetable–rich diets are associated with lower BP and lower risk of ischemic stroke and ischemic heart disease.^69^, ^70^, ^71^ Although these effects have been demonstrated in many populations, the exact mechanism of this protection remains unknown. Epidemiological studies that have sufficient numbers of persons and adequate dietary data to examine the protective effects of various *categories* of fruits and vegetables find that green leafy vegetables appear to confer the highest degree of protection against cardiovascular disease.^69^ Green leafy vegetables are also the major source of nitrate in the diet of most Americans.^72^

There is ecological evidence for a hypothesis that the cardioprotective benefit of vegetables may be conferred by nitrate. For example, a traditional Japanese diet is very high in nitrate from vegetables, and Japan has historically lower rates of coronary heart disease than the United States.^73^ In contrast, India, a country with a high incidence of coronary heart disease, has low levels of nitrate in the traditional diet, similar to a typical US diet.^74^ Usual dietary consumption of nitrate in the United States is estimated to be between 40 and 100 mg/day, with nitrite ingestion at much lower levels (0 to 20 mg/day).^72^ Dietary patterns associated with BP lowering, such as the Dietary Approaches to Stop Hypertension diet, used vegetable combinations with ≈160 mg of nitrate/day^75^ and could contain dietary nitrate concentrations ranging from 174 to 1222 mg/day depending upon whether low‐ or high‐nitrate food choices were used.^72^ As such, it is biologically plausible that habitual dietary nitrate concentration may, in part, explain population risk for cardiovascular disease.

Although 80% to 85% of nitrate is derived from vegetables, nitrite is obtained mostly through cured meats in the diet. For centuries, meats have been preserved using nitrite to protect against botulism and to confer a red color to meat. Responding to concerns about the potential for formation of N‐nitrosamines, US food regulations in 1978 and 1986 lowered the level of residual nitrite allowable in processed meat.^76^ These revisions succeeded in substantially lowering nitrite level in cured meat: Surveys during 1971–1972 yielded mean nitrite levels between 26 and 64 parts per million (ppm)^77^; by 2012, mean nitrite in US cured meat products had declined to a range of 0.1 to 11 ppm.^78^ In a 1975 study, notwithstanding the higher levels of nitrite in processed meat of that era, White calculated that 80% of nitrite that entered the stomach was derived from saliva, rather than directly from the diet.^77^

To better assess the relation between dietary nitrate and nitrite and cardiovascular disease, cohort studies with both accurate dietary nitrate assessment and cardiovascular event characterization are needed. Two studies that collect dietary and health data from a large, geographically diverse sample of the US population were reviewed at the current workshop. The Reasons for Geographic and Racial Differences in Stroke (REGARDS) Study and The National Health and Nutrition Examination Surveys (NHANES) may offer opportunities to study the epidemiological relation between dietary nitrate intake and coronary risk and outcomes. REGARDS is following 30 239 black and white participants age 45+, sampled from residents in 60.3% of US counties.^79^ Follow‐up for cardiovascular case ascertainment is conducted by means of hospitalization abstractions and death certificate examination. NHANES assesses the health and nutritional status from a cross‐sectional sample of adults and children in the United States, combining personal interviews with standardized physical examinations and laboratory tests.^80^ Every year, a nationally representative sample of ≈5000 individuals of all ages completes the survey. There is no follow‐up for subsequent clinical events, but, annually, all current and past survey participants are matched with the National Death Index to ascertain eventual cause of death. In addition, NHANES data are linked to Medicare administrative records so that information on cardiovascular events among older adults may also be examined.

However, exposure estimates for dietary nitrate and nitrite in cohorts such as these remain difficult because of the lack of widely applicable dietary databases that include the nitrate and nitrite content of common foods.^81^ Compared to other dietary nutrients, there can be substantial variability in nitrate composition between samples of the same vegetable species. During growth, the plant takes up nitrate from the roots and accumulates varying amounts of nitrate in different parts of the plant. Overall, nitrate flux in plants is controlled both by internal nutritional status and by external abiotic factors, including water, light, and external nitrate concentration. Variability in nitrate content of a consumed vegetable can result from varietal differences, differences in growing temperature and amounts of rainfall or irrigation, the amount of nitrogen‐containing fertilizers or amount of nitrate in irrigation water, and differences in postharvest storage and handling.^82^ Such factors of variability will, for example, inevitably result in vastly different nitrate content of any 1 vegetable from 1 farm to another let alone from 1 state to another. Analyses that evaluate health outcomes within any cohort as a function of habitual dietary nitrate ingestion may need to incorporate contemporaneous measures of this variability when assessing the nitrate composition of dietary components.

## Nitrite and Infant Methemoglobinemia

Notwithstanding the likely cardiovascular benefits of nitrate from dietary vegetables, high levels of dietary nitrate or nitrite can be problematic for very young infants. In the newborn, both serum nitrate and nitrite are at very low concentrations for the first few weeks of their lives.^83^ It is well established that excessive gastrointestinal nitrite absorption puts young infants at risk of methemoglobinemia (met‐Hb) because of a neonate's high proportion of fetal hemoglobin and relative lack of metHb‐reducing capacity.^84^ Very young infants, with their undeveloped oral microbiome, do not produce nitrite from salivary nitrate.^83^ But preformed nitrite from improperly stored vegetable purees fed to young infants, or nitrite formed in their stomach from overgrowth of nitrate‐reducing bacteria, can cause MetHb. The higher pH of a young infant's stomach allows its colonization by nitrate‐reducing bacteria usually found in the lower gastrointestinal tract,^84^ thereby increasing the likelihood of excessive nitrite absorption when high‐nitrate vegetable purees are fed or when high‐nitrate water is used to prepare infant feeding formula.^85^, ^86^, ^87^, ^88^ This “well‐water cyanosis” has been found to occur only in the first few weeks of life, in infants with gastric pH >4, and in whom the upper gastrointestinal tract has been populated by nitrate‐reducing organisms.^89^

Contrary to the situation for absorbed nitrite healthy infants appear to be quite tolerant of orally ingested nitrate in the absence of bacterial contamination or coinfection. In fact, some commercially prepared infant formulas have been found to exceed the World Health Organization recommended standard for adult nitrate exposure.^90^ A review conducted by the Canadian Food and Drug Directorate cited studies in which healthy infants as young as 3.5 to 8 months, when fed high‐nitrate vegetables, absorbed the nitrate rapidly in the upper gastrointestinal tract and excreted them in the urine.^91^ This review concluded, however, that because of their unique physiology and predilection to upper gastrointestinal bacterial colonization, infants younger than 3 months of age should be prevented from ingesting high‐nitrate food or water.^91^

## Exogenous Nitrate From Drinking Water

In recognition of the enhanced risk for young infants, the US maximum contaminant level of 10 mg/L of nitrate‐N (44 mg/L nitrate) for drinking water was established in 1975.^92^ Whereas nitrite is implicated in the etiology of methemoglobinemia, nitrite concentrations are typically observed to be low in drinking water, unless bacterial contamination is present. Nitrate, in contrast, may occur at high levels in drinking water, particularly in agricultural areas that receive high input from nitrogen‐based fertilizers, mostly applied as anhydrous ammonia or ammonium compounds. In US groundwater supplies, water exceeding the maximum contaminant level for nitrate is found mostly in private well water in rural areas. For persons drinking water that is above 11.3 mg/L of nitrate‐N, ie, slightly higher than the maximum contaminant level, the nitrate contribution from drinking water will usually exceed that from vegetable intake.^93^ A national study indicated that 22% of private wells in US agricultural areas exceed the nitrate maximum contaminant level of 10 mg/L of nitrate‐N.^94^ When ammonia (NH_3_) fertilizers are applied to soil, ammonia is readily converted to nitrate by the action of soil microbes; this nitrate percolates into the local groundwater. Nitrate in groundwater has increased in the past 50 years primarily because of the application of fertilizers and is highest in areas with high nitrogen input, irrigation, and well‐drained soils or fractured rocks. Vertical rates of water movement in the aquifer and the span of ages of water entering the well also affect nitrate concentration in sampled wells. All of these variables have been incorporated into models that are improving our understanding of factors that influence nitrate in groundwater and the geographical distribution of areas with high levels of groundwater nitrate.^95^, ^96^

## Chronic High Intake of Nitrate or Nitrite and Possible Carcinogenicity

Although the concern regarding young infants has always focused on the acute toxicity of nitrite and nitrate, for the remainder of the population, the issue has been the possible effects of sustained high levels of ingestion, particularly in drinking water, that is, nitrate consumed in the absence of an antioxidant‐rich plant matrix. Assuming that the nitrate has been reduced to nitrite either preingestion or postingestion by the enterosalivary cycle, when swallowed nitrite arrives in the stomach, rather than being reduced to nitric oxide, it may alternatively enter an N‐nitrosation pathway. The nitrite, now acidified to nitrous acid (HNO_2_^−^), can react with dietary amines and amides to form carcinogenic N‐nitroso compounds.^97^, ^98^, ^99^

It has been demonstrated that endogenous nitrosation occurs in humans dosed with inorganic nitrate and an amine source,^100^, ^101^, ^102^ but also that this reaction is inhibited by concurrent ingestion of food matrix components, for example, polyphenols, ascorbate (vitamin C), or α‐tocopherol (vitamin E).^100^, ^103^, ^104^, ^105^ Healthy persons have levels of ascorbate in their gastric juices that are several times higher than in plasma, indicating active secretion of ascorbate into the stomach, a process that may confer some protection against gastric nitrosation of dietary components.^106^ In the US, food safety regulations require that either ascorbate or erythorbate, an isomer of ascorbate, be added to all processed meats containing nitrite preservatives.^107^

Any adverse health effects attributed to nitrate and nitrite ingestion would result from a complex interaction of the amount of nitrate and nitrite ingested, the acidity of the stomach, concomitant ingestion of food matrix components that decrease the potential for nitrosation chemistry, smoking status, and specific medical conditions that increase nitrosation.^99^ Gastric nitrosation is maximized at low pH,^108^ implying that if N‐nitrosamines are involved in the initiation of stomach cancer, that initiation likely precedes the onset of gastric lesions that would themselves increase the pH of the gastric lining.^109^

The International Agency for Research on Carcinogenicity (IARC) found that epidemiological evidence for an association between dietary nitrate or nitrite and human cancer is strongest for *nitrites* and *stomach cancer*.^109^ In their review of animal studies, they also found “sufficient evidence in experimental animals for the carcinogenicity of nitrite in combination with amines or amides.”^109^ When the IARC turned its attention to dietary *nitrate* and risk of stomach cancer, however, the only significant associations found among 9 large population studies^110^, ^111^, ^112^, ^113^, ^114^, ^115^, ^116^, ^117^, ^118^, ^119^, ^120^, ^121^ were *inverse* associations between dietary nitrate and stomach cancer detected in 4 of the case‐control studies, an association likely conferred by the protective components present in fruits and vegetables.^112^, ^114^, ^115^, ^116^, ^117^

The 2010 IARC report summarized with the determination that: “Ingested nitrate or nitrite *under conditions that result in endogenous nitrosation* is probably carcinogenic to humans.”^109^ The current workshop members, in turn, expressed the opinion that future research on nitrate and nitrite carcinogenicity should focus on subpopulations with increased risk of endogenous N‐nitrosation of amines, such as smokers or persons consuming high doses of nitrate or nitrite as supplements or low amounts of food matrix constituents, such as polyphenols and essential nutrients, that may decrease endogenous nitrosation reactions in the stomach.

## Summary and Recommendations

Research in recent decades has demonstrated that the body can use exogenous nitrite and nitrate to produce the important messenger molecule, nitric oxide. The cardiovascular protective effect of a diet rich in fruits and vegetables may be attributed, in part, to its high nitrate content, contributed largely by leafy green vegetables and root vegetables. Currently, evidence suggests that a diet naturally rich in fruits and vegetables may offer the cardiovascular benefits of enhanced nitric oxide production while conferring protection from formation of N‐nitrosamines. High levels of nitrate in drinking water or dietary supplements are of more concern because of possible isolated ingestion without the protective dietary components found in a complex vegetable matrix. Further research assessing these issues would be of value.

The working group made the following recommendations for advancing knowledge of the relation between dietary nitrate and cardiovascular disease in populations:

1. Improve the standard dietary databases used in epidemiological research to include concurrent and where possible “local” estimates of the nitrate content of commonly eaten vegetables, such that when dietary patterns are evaluated for cardiovascular benefit, the nitrate content is taken into account. Large cohort studies should incorporate measures of nitrate in urine and plasma whenever possible to ensure that estimates of ingestion are associated with empirical measures of exposure.
2. Add measures of drinking water nitrate content to observational cohort data collection, particularly those that include persons in rural areas drinking from private wells. Although these persons are a minority of the US public, it is important to accurately ascertain whether they are at increased risk of cancer resulting from chronic high water nitrate ingestion without simultaneous ingestion of food components that may decrease risk of gastric nitrosation, and whether they experience any long‐term cardiovascular benefit from their high level of nitrate ingestion.
3. Current research in nitrate carcinogenicity does not support a role for nitrate consumed as vegetables in the diet. Future carcinogenicity research should focus on subpopulations at increased risk for endogenous N‐nitrosamine formation (eg, smokers, or persons consuming high levels of nitrate or nitrite, such as through supplement ingestion) in the absence of protective food matrix components, such as polyphenols or essential nutrients.

## Sources of Funding

The workshop was convened and funded by the National Heart, Lung, and Blood Institute.

## Disclosures

Ahluwalia is a Director of Heartbeet Limited. Kim‐Shapiro is listed as a co‐inventor on a patent related to use of nitrite in cardiovascular conditions and owns stock in and serves on the scientific advisory board for Beverage Operations LLC, which has licensed Wake Forest University intellectual properties and thus has a financial interest in Beverage Operations LLC. Lefer is a participant of a pending US patent filed on October 14, 2003 (Patent No. 60/511244) regarding the use of sodium nitrite in cardiovascular disease. Lefer is a participant of a pending US patent filed on November 15, 2007 (Patent No. 61/003150) regarding the use of nitrite salts in chronic ischemia. Schechter and Gladwin are co‐inventors of patents to the National Institutes of Health, which have been licensed for development to several companies, for the potential use of nitrite ions for treating cardiovascular diseases.
